# Immune Checkpoint Inhibitors for the Treatment of Central Nervous System (CNS) Metastatic Disease

**DOI:** 10.3389/fonc.2018.00414

**Published:** 2018-09-27

**Authors:** Suneel D. Kamath, Priya U. Kumthekar

**Affiliations:** ^1^Northwestern Medicine, Chicago, IL, United States; ^2^Feinberg School of Medicine, Northwestern University, Chicago, IL, United States; ^3^Robert H. Lurie Comprehensive Cancer Center of Northwestern University, Chicago, IL, United States

**Keywords:** immunotherapy, brain metastasis, CNS metastasis, checkpoint inhibitors, PD-1, pembrolizumab, nivolumab, ipilimumab

## Abstract

While the CNS has long been viewed as an immune-privileged environment, a paradigm shift in neuro-immunology has elevated the role of systemic immunotherapy for the treatment of metastatic disease. Increasing knowledge regarding the presence of a CNS lymphatic system and the physical and biochemical alteration of the blood brain barrier (BBB) by the tumor microenvironment suggests immune cell trafficking in and out of the CNS is possible. Emerging clinical data suggest immune checkpoint inhibitors (ICIs) can stimulate T cells peripherally to in turn have anti-tumor effects in the CNS. For example, anti-programmed cell death-1 (PD-1) monotherapy with pembrolizumab has shown intracranial response rates of 20–30% in patients with melanoma or non-small cell lung cancer (NSCLC) brain metastases. The combination of nivolumab and ipilimumab [anti-PD-1 and anti-cytotoxic T-lymphocyte-associated protein 4 (CTLA-4)] showed an intracranial response rate of 55% in patients with melanoma brain metastases. More data are needed to confirm these response rates and to determine mechanisms of efficacy and resistance. While local therapies such as stereotactic radiosurgery (SRS), whole-brain radiation therapy (WBRT), and surgery remain current mainstays, ICIS offer potential decreased neurotoxicity. This review summarizes the biological rationale for systemic immunotherapy to treat CNS metastatic disease, existing clinical data on ICIs in this setting and ongoing clinical trials exploring areas of unmet need.

## Introduction

Despite recent advances in cancer therapy, CNS metastasis remains a devastating complication for many solid organ cancer patients. Brain metastases occur in up to 20% of adults with systemic malignancies, most commonly in lung cancer, melanoma, and breast cancer (1). The incidence is increasing in many histologies, in part due to improved detection by magnetic resonance imaging (MRI) and with prolonged survival from improved systemic therapies (2). To date, local therapies such as SRS, WBRT, and surgical resection have been the mainstays. These modalities can cause significant complications and morbidity (stroke, radiation necrosis, cognitive deficits) with only a modest benefit in overall survival (3).

Systemic immunotherapy has shown promising early results in treating brain metastases and has altered the traditional immune-privileged paradigm of the brain. The immune system plays an important role in clearance of oncogenic clones through antigen-presenting cell (APC) recognition of tumor cell antigens, T cell activation by APCs and subsequent T cell cytotoxicity (4, 5). Conversely, tumor cells can evade immune destruction through expression of various immune checkpoints that promote self-tolerance and suppress effector T cell function and proliferation (6, 7). The most clinically relevant immune checkpoints are programmed cell death protein 1 and its ligand (PD-1 and PD-L1) and cytotoxic T-lymphocyte-associated protein 4 (CTLA4). PD-L1 is expressed on the tumor cell surface and through interaction with PD-1 on T cells causes apoptosis of cytotoxic T cells while inhibiting apoptosis of regulatory T cells (8). CTLA4 is a co-stimulatory pathway protein that interacts with HLA-B7-1 and HLA-B7-2 on T cells and delivers an inhibitory signal to effector T cells while promoting inhibitory function of regulatory T cells (6–8). In net, these pathways promote tumor cell survival and proliferation through immune evasion.

While normal brain parenchyma and primary CNS tumors have immunoregulatory environments with rare lymphocytes, brain metastases have been shown to have significant tumor-infiltrating lymphocytes (TILs). One series of 116 patients with brain metastases specimens showed that CD3+ TILs were present in 115/116 (99.1%) specimens and 56% had dense or very dense TIL infiltration (9). 112/116 (96.6%) tumor specimens had CD8+ T cell infiltration while 19/67 (28.4%) of specimens evaluated for PD-L1 expression had > 5% membranous expression. The highest density of CD3+ TILs, CD8+ TILs, and PD-1-expressing T cells was found in melanoma brain metastases. High density of CD3+ TILs was associated with longer median overall survival (OS) regardless of primary tumor site compared to low CD3+ TIL density (15 months vs. 6 months, respectively) (9). It has previously been demonstrated that higher density of TILs, CD8+ T cells, and CD45RO+ memory T cells in the primary tumor is associated with longer disease-free survival and OS in various solid tumor cancers (4). The concordance of higher TIL density and improved OS in both primary tumors and brain metastases supports the use of immune checkpoint inhibition to treat both systemic and CNS metastatic disease. Given that the brain is no longer a strict “immune privileged” environment and brain metastases can disrupt the blood brain barrier, there have been several trials of ICIs with promising results that are summarized herein.

## Clinical data

### Ipilimumab

The anti-CTLA4 monoclonal antibody ipilimumab was the first ICI to show efficacy in treating brain metastases. This CNS activity was discovered incidentally in the original trials establishing its efficacy in metastatic melanoma when patients with brain metastases also showed durable CNS responses (10). This was confirmed in a subsequent phase II study of 72 patients with melanoma brain metastases treated with ipilimumab 10 mg/kg every 3 weeks (11). Of 51 patients with asymptomatic brain metastases, 16% showed an objective response in the CNS with a median CNS progression-free survival (PFS) of 1.5 months. Of 21 patients with symptomatic brain metastases or those that required steroids, the objective response rate (ORR) was 5% with median CNS PFS of 1.2 months. CNS disease control was achieved in 24% of asymptomatic patients and 10% of symptomatic patients (11). A second phase II trial (NIBIT-M1 trial) of ipilimumab 10 mg/kg combined with fotemustine in metastatic melanoma showed a 40% CNS response rate, though only 20 patients with brain metastases were included in this trial. Two patients achieved a CNS complete response (CR) and 50% of patients achieved CNS disease control (12). OS at 3 years was 27.8%, suggesting that responses and disease control were durable in this population as has been observed in other immunotherapy trials (13).

An expanded access program in the United States that included 165 patients with melanoma brain metastases treated with ipilimumab demonstrated a 20% rate of OS at 1 year (14). Another expanded access program in Italy that included 146 patients with melanoma brain metastases treated with ipilimumab demonstrated an ORR of 12% and disease control rate of 27%. This included 4 patients who achieved a CR (15). Of note, these trials used high-dose ipilimumab, which is associated with a higher rate of severe colitis and treatment-related mortality (16, 17). As a result, the 10 mg/kg dose is uncommon in more recent clinical trials, especially when dual immunotherapy is used, where the 1–3 mg/kg doses are often used (18, 19). Overall, these data were the first to establish efficacy and durability of ICIs for the treatment of CNS metastatic disease and paved the way for anti-PD-1 therapy in this setting.

### Pembrolizumab

The anti-PD-1 monoclonal antibody pembrolizumab was the first PD-1 inhibitor that clearly demonstrated efficacy against untreated brain metastases in melanoma and non-small cell lung cancer (NSCLC). The original trial was a single-institution, phase II trial that included 2 cohorts of patients with untreated or progressive brain metastases, one for melanoma and one for NSCLC (17). All levels of PD-L1 expression were included in the melanoma cohort and a cutoff of ≥ 1% was used in the NSCLC cohort. In the 18 patients with melanoma brain metastases, 4 (22%) patients experienced an objective response, 2 CRs and 2 partial responses (PRs). An additional 4 patients had stable disease. In the NSCLC cohort of 18 patients, 6 (33%) had an objective response, which included 4 CRs and 2 PRs. One patient achieved stable disease as the best response (17).

There was strong concordance between CNS response and systemic response as 8/9 (88%) of patients with a confirmed systemic response also had a CNS response. After 11.6 months of follow up, median OS in the melanoma cohort was not reached and was 7.7 months in the NSCLC cohort. The toxicity profile was similar to other trials of pembrolizumab across disease types and importantly, there were no treatment-related deaths. Of note, pembrolizumab was given as 10 mg/kg every 2 weeks in this trial as opposed to the fixed dose of 200 mg every 3 weeks that is FDA-approved now (17).

An update of data for the NSCLC cohort presented at the ASCO 2018 annual meeting showed a CNS response rate of 29.4% in the 34 patients enrolled (20). Median OS was 8.9 months with 31% of patients living more than 2 years. Discordance between CNS and systemic responses was seen in 7 patients (21%, 4 with CNS disease progression but PR systemically and 3 with CNS response but systemic disease progression). An additional 5 patients with PD-L1 negative or unevaluable tumors were included, though there were no CNS responses in this subgroup (20).

### Nivolumab

The anti-PD-1 monoclonal antibody nivolumab has shown similar efficacy to pembrolizumab for untreated melanoma brain metastases. In the monotherapy arm of the randomized phase II ABC study, patients with asymptomatic melanoma brain metastases treated with nivolumab 3 mg/kg every 2 weeks showed a 20% CNS response rate (21). Median intracranial PFS and OS were 2.5 and 18.5 months, respectively. In a cohort of patients with symptomatic brain metastases, leptomeningeal disease or failure of local radiotherapy, a 6% CNS response rate was observed (21).

Additional data supporting nivolumab for untreated brain metastases comes from an Italian expanded-access program of 372 patients with advanced squamous NSCLC, 38 of whom had asymptomatic brain metastases (22). The disease control rate was 47.3% in this cohort, comprising of one CR, six PRs and 11 patients with stable disease. Four patients were treated beyond progression. The median PFS and OS were 5.5 and 6.5 months, respectively and only 1 patient discontinued therapy due to adverse events (22). Another small series of 5 patients with asymptomatic NSCLC brain metastases treated with nivolumab showed activity in 3 patients: 1 CR, 1 PR, and 1 with stable disease for 10 weeks. Both responses were durable beyond 6 months (23).

Another Italian expanded access program for nivolumab in metastatic renal cell carcinoma (RCC) included 389 patients, 32 with brain metastases. The CNS response rate was 18.7% with a disease control rate of 53.1%. This included 1 CR, 5 PRs, and 11 with stable disease. The CNS response rate was similar to the systemic response rate of 23.2%. The 1 year OS rate was 63.1% and in a univariate analysis, CNS metastasis was not associated with inferior OS (24).

### Nivolumab and ipilimumab

The combination of nivolumab and ipilimumab has shown the most impressive CNS response rates to immunotherapy. The phase II CheckMate 204 study of nivolumab 1 mg/kg and ipilimumab 3 mg/kg every 3 weeks was the first to demonstrate efficacy of combination immunotherapy for patients with untreated melanoma brain metastases (25). An updated analysis of CheckMate 204 with 94 enrolled patients showed a 52% CNS response rate, including 24 (26%) intracranial CRs (26). The intracranial clinical benefit rate was 57%. The systemic ORR was 47% with high concordance between systemic and CNS responses. Only 5 patients (5%) discontinued therapy due to immune-related neurologic adverse events, though there was one death due to immunotherapy-related myocarditis.

These results were confirmed in the randomized phase II ABC study comparing nivolumab 3 mg/kg every 2 weeks alone vs. combination therapy with nivolumab 1 mg/kg and ipilimumab 3 mg/kg every 3 weeks (21). This study showed a CNS response rate of 46% with the combination of nivolumab and ipilimumab vs. 20% with nivolumab alone. This included CNS complete response rates of 17 and 12%, respectively. Median intracranial PFS and median OS were both not reached in the combination therapy arm after a median follow up of 14 months (21).

The combination of nivolumab and ipilimumab was significantly more toxic than nivolumab alone. Grade 3 or higher adverse events were observed in 63% of patients receiving the combination vs. 16% receiving nivolumab alone and were mainly systemic. There was only one grade 3 CNS adverse event that was more common with combination therapy vs. nivolumab monotherapy, which was headache (20 vs. 6%). There were no treatment-related deaths in this trial (21).

Overall, these data demonstrate that the combination of anti-PD-1 and anti-CTLA4 therapy has significant activity for CNS metastatic disease with a relatively low rate of serious CNS-specific toxicity. Larger randomized clinical trials are needed to confirm these findings and to identify predictive biomarkers for CNS response. One such study, the phase III NIBIT-M2 study randomizing patients with melanoma brain metastases to fotemustine, fotemustine, and ipilimumab or ipilimumab and nivolumab, is ongoing (27).

A table summarizing the clinical trials discussed is shown in Table 1.

**Table 1:** Summary of immunotherapy trials for CNS metastatic disease.

**Trial**	**Drug(s)**	**Phase**	***N* (ITT)**	**Disease**	**PD-L1 status**	**CNS ORR**	**Median CNS PFS (months)**	**Median PFS (months)**	**Median OS (months)**
NCT00623766	Ipilimumab 10 mg/kg q3W × 4 doses, then 10 mg/kg q12W	2	51	Melanoma (asymptomatic BMs)	NA	16% (8/51)	1.5	1.4	7
			21	Melanoma (symptomatic BMs or on steroids)	NA	5% (1/21)	1.2	1.2	3.7
NIBIT-M1	Ipilimumab 10 mg/kg q3W × 4 doses, then 10 mg/kg q12W + fotemustine 100 mg/m2 q3W	2	20	Melanoma (asymptomatic BMs)	NA	40% (8/20)	3	4.5	13.4
NCT02085070	Pembrolizumab 10 mg/kg q2W	2	18	Melanoma	Any	22% (4/18)	not reported	not reported	NR
			18	NSCLC	≥ 1%	33% (6/18)	not reported	not reported	7.7
CheckMate 204	Nivolumab 1 mg/kg q3W + Ipilimumab 3 mg/kg q3W	2	75	Melanoma	Any	56% (42/75)	not reported	not reported	not reported
NCT02374242	Nivolumab 1 mg/kg q3W + Ipilimumab 3 mg/kg q3W	2	35	Melanoma (asymptomatic BMs)	Any	46% (16/35)	NR	13.8	NR
	Nivolumab 3 mg/kg q2W		25	Melanoma (asymptomatic BMs)	Any	20% (5/25)	2.5	2.6	18.5
	Nivolumab 3 mg/kg q2W		16	Melanoma (symptomatic BMs, failed local therapy)	Any	6% (1/16)	2.3	2.6	5.1

*N, number; ITT, intention to treat; W, week; NA, not applicable; BMs, brain metastases; NR, not reached*.

### Additional case series

There are several single-institution series of ICIs for patients with CNS metastatic disease. One series from Cleveland Clinic included 128 patients with brain metastases from NSCLC (94 patients), RCC (15 patients), or melanoma (19 patients) who were treated with either pembrolizumab, nivolumab, ipilimumab, or a combination. Patients could also receive WBRT or SRS. While the authors did not report on CNS response or disease control rates, they reported 1 year survival rates of 48.3, 54.5, and 55.4% in patients with NSCLC, melanoma, and RCC, respectively (28).

Another single-institution series from the University of Cincinnati identified 51 patients with brain metastases from NSCLC, small cell lung cancer (SCLC), melanoma and head, and neck squamous cell carcinoma (HNSCC). Thirty patients had symptomatic brain metastases. Patients were treated with either atezolizumab, durvalumab, pembrolizumab, nivolumab, ipilimumab, or a combination. They could also receive concurrent radiation. The authors did not report CNS response or disease control rates, but reported median OS after the start of immunotherapy of 7.6, 7.2, 6.2, and 4 months for patients with melanoma, NSCLC, SCLC, and HNSCC, respectively. They also found that patients treated with immunotherapy alone had worse survival compared to combined modality therapy with radiation or surgery (29).

### Future directions and challenges

Immunotherapy in neuro-oncology is an active area of investigation given its potential efficacy and clinical impact. Since most patients with brain metastases receive radiation therapy at some point, understanding the interplay of radiation with immunotherapy is of particular interest. Historically, radiation was considered to be immunosuppressive due to peripheral blood lymphodepletion (30). More recent pre-clinical work has shown that radiation can augment anti-tumor immune responses through several mechanisms. First, radiation-induced tumor cell necrosis increases the release of tumor neoantigens and increases tumor mutational burden (TMB) (31). This also triggers the release of immune-stimulatory damage-associated molecular patterns (DAMPs), including high-mobility group protein B1 (HMGB1) and calreticulin (32, 33). These promote APC recruitment to the tumor microenvironment and antigen uptake and presentation to cytotoxic T cells. Radiation primes CD8 T cells by stimulating IFN-γ production and increasing tumor cell MHC class I and Fas expression (34). Radiation also increases PD-L1 expression, creating an opportunity for synergy with anti-PD-1 therapy (31, 35).

Small series have shown synergy between ipilimumab and SRS. One series of 70 patients with melanoma brain metastases showed improved median OS in 37 patients who received ipilimumab and SRS vs. the 33 patients who received SRS alone (18.3 months vs. 5.3 months) (36). Another series of 77 patients (27 received ipilimumab and SRS, 50 received SRS alone) also showed improved median OS of 21 months vs. 5 months, respectively (37). It appears that concurrent radiation with checkpoint inhibition is more effective than sequential treatment and high-dose, hypofractionated radiation is best, but this must be confirmed with more clinical data (31, 38). Steroid administration during SRS can negatively impact response to ICIs and must be considered only when clinically necessary (39).

Radiation delivered to a local site has been shown to cause regression of distant metastatic sites outside of the radiation field, a phenomenon known as the abscopal effect (32, 40). Activated cytotoxic T lymphocytes from increased tumor antigen stimulation and presentation at a local tumor site are thought to mediate the effect seen at distant tumor locations (32, 34, 40). Immune checkpoint inhibition dramatically improves the abscopal effect of radiation in pre-clinical models (32, 34). There are also two case reports (one NSCLC and one melanoma) of patients who achieved durable systemic complete responses at all tumor sites for 1 year with concurrent ipilimumab and local site stereotactic body radiation therapy (41, 42).

Based on these promising results, there are numerous ongoing clinical trials combining ICIs and brain radiation (NCT03104439, NCT02608385, NCT02730130, NCT03453164, NCT02444741), (NCT02696993).

Despite the fact the CNS is no longer considered an immune privileged site, it remains at least an immune deficient environment. While TILs have clearly been identified in CNS metastases, they are in lower number than in systemic tumors and the ratio of effector T cells to regulatory T cells remains unknown (9, 43). T cell and APC trafficking into and out of the CNS is more strictly regulated than in other tissues (9, 44). The degree of blood brain and blood tumor barrier disruption is variable between diseases, patients and even individual lesions in the same patient (45–47).

Patients with brain metastases frequently require steroids for symptomatic control, which has been found to negate the mechanisms of immunotherapy. In a series of 244 metastatic NSCLC patients, the use of steroids at > 20 mg/day of prednisone was associated with worse median PFS (1 month vs. 3 months) and median OS (3 months vs. 10 months) (48). Only 19 patients received > 20 mg/day of prednisone and it remains unclear if high-dose steroids truly blunt the effect of immunotherapy or simply select for a population with worse overall prognosis (48).

A substantial number of patients do not respond systemically or in the CNS to existing immunotherapy drugs, creating a tremendous unmet need. The combination of pembrolizumab and bevacizumab is being studied in a phase II clinical trial in patients with untreated NSCLC and melanoma brain metastases (NCT02681549). The anti-PD-L1 drug atezolizumab is also being studied in combination with bevacizumab for untreated melanoma brain metastases (BEAT-MBM study; NCT03175432). This study includes a cohort with symptomatic brain metastases or those who require corticosteroids. Indoleamine (2,3)-dioxygenase (IDO) has emerged as another immune checkpoint that can be combined with anti-PD-1 therapy. There is an ongoing phase II study evaluating the IDO inhibitor BMS-986205 in combination with nivolumab for untreated melanoma brain metastases. A phase II trial evaluating pembrolizumab for patients with leptomeningeal carcinomatosis is ongoing, but to date, there have been no completed randomized trials in this population (49). Data from these trials and others may further expand the role of immunotherapy for the treatment of CNS metastatic disease.

There is also a significant need to identify more predictive immune biomarkers in the CNS. Many series have shown increased density and/or number of CD3+ and/or CD8+ TILs in brain tumor specimens is correlated with improved survival (9, 43, 50, 51). It is important to note that TIL density is lower in general in brain metastases and discordance in TIL density between primary tumors and brain metastases may be as high as 48% (51, 52). PD-L1 expression (≥ 1%) is present in ~ 25–30% of brain tumor specimens in some series, but it may be discordant from primary tumors in 30% of cases (9, 51, 53). Discordance in TIL density or PD-L1 expression can be partially explained by temporal and spatial heterogeneity from biopsies taken at different time points and from different sites (51). The predictive value of PD-L1 expression was shown in one series in which NSCLC patients with PD-L1+ brain metastases had a 29% intracranial response rate, while those with PD-L1- negative brain metastases had no responses (54). However, the data overall are very limited and prospective validation is still required. High TMB in brain metastases has been reported in 39% of cases and was more common than in primary tumors in one series (55). This may emerge as a clinically useful biomarker in the future.

As evidenced by the trials reviewed herein, there are disparate primary outcomes and disease measures, creating a need for consistent and clear CNS-specific endpoints in these studies. For example, the phenomenon of pseudo-response seen with the vascular endothelial growth factor (VEGF) inhibitor bevacizumab may alter a study whose primary outcome is response rate (56). Furthermore, response is seen less often in the brain than systemically and using clinical benefit as an endpoint might be more accurate. These are all considerations that need to be unified across brain metastases clinical trials (56, 57).

## Conclusions

Patients with brain metastases have traditionally been excluded from clinical trials, which is a detriment to our understanding of systemic therapies for CNS metastatic disease. A 2014 systematic analysis of interventional drug trials in advanced NSCLC listed on www.ClinicalTrials.gov showed that only 26% allowed for patients with untreated brain metastases (58). An ongoing analysis of currently available trials for advanced NSCLC showed only 27.7% specifically allowed enrollment of patients with untreated asymptomatic brain metastases and only 3.7% included patients with symptomatic or progressive brain metastases. While these patients have often been excluded because of lacking pre-clinical data or concerns about worsening outcome measures, it is important that we include these patients as they are more representative of the real-world disease population (59).

While there are legitimate barriers for clinical trial design and patient enrollment, the early data for immunotherapy in CNS metastatic disease show some promise and necessitate more studies where brain metastases are not exclusionary criteria. This viewpoint is further supported by the American Society of Clinical Oncology—Friends of Cancer Research Brain Metastases Working Group recommendation statement from November 2017 (59). Their recommendations provide a clear and practical framework to improve clinical trial eligibility criteria for patients with brain metastases without compromising good scientific trial design.

As systemic therapies improve and patients live longer with metastatic disease, the number of patients with CNS metastases will grow, creating a larger unmet need for cancer patients. The existing evidence of the efficacy of systemic immunotherapy for untreated brain metastases is promising and supports increased enrollment of patients with brain metastases in immunotherapy clinical trials.

## Author contributions

SK and PK both contributed equally to the conception, design, writing, and editing of this manuscript as well as the selection and construction of its figures/tables.

### Conflict of interest statement

The authors declare that the research was conducted in the absence of any commercial or financial relationships that could be construed as a potential conflict of interest.
